# Comparing the effects of *Cyperus esculentus* hydroethanolic extract and *Euterpe oleracea* on reproductive efficacy against cadmium-induced testicular toxicity in male rats

**DOI:** 10.5455/javar.2023.j724

**Published:** 2023-12-27

**Authors:** Sura Safi Khafaji

**Affiliations:** Department of Physiology, Biochemistry, and Pharmacology, College of Veterinary Medicine, Al-Qasim Green University, Al-Qasim City, Babylon Province, Ministry of Higher Education and Scientific Research, Iraq.

**Keywords:** Cyperus esculentus, cadmium chloride, Euterpe oleracea, gene expression, oxidative stress, reproductive toxicity

## Abstract

**Objective:**

Cadmium chloride (CdCl_2_) is an environmentally toxic pollutant that can cause reprotoxicity. *Cyperus esculentus* and* Euterpe oleracea* are potent antioxidant plants currently used to counteract the action of harmful pollutants. The present experiment was intended to evaluate and comp are the role of *C. esculentus* hydroethanolic extract (CHE) and *E. oleracea* in treating the reprotoxicity induced by CdCl_2_ in rats.

**Materials and Methods:**

Forty adult male rats (160–210 gm) were allocated into five groups equally. Control group: received 5 ml of normal saline (NS); the other treatment groups were injected with CdCl_2_ as a single dose for two weeks to induce testicular toxicity. After 14 days, the four groups were treated orally daily for two months as follows: The cadmium group (Cd) received NS, the third group (TC) was administered 800 mg/kg BW of CHE, the fourth group (TO) received 500 mg/kg BW of* E. oleracea,* and the fifth group (TCO) received CHE with* E. oleracea.*

**Results:**

The live sperm and motility, serum testosterone, follicle-stimulating hormone (FSH), testicular superoxide dismutase (SOD), catalase (CAT), steroidogenic acute regulatory protein (StAR), 17*β*-hydroxysteroid dehydrogenase, and 3β-hydroxysteroid dehydrogenase (3*β*-HSD) were significantly increased in the TCO, TC, and TO groups compared with the Cd group. Testicular nitric oxide and malondialdehyde were elevated significantly in the Cd group compared to the TC, TO, TCO, and control groups. The fold changes of *Fshβ*, *Lhβ*, and *Gnrh* genes were upregulated in the TCO group compared to the Cd and control groups.

**Conclusion:**

The combination of CHE with* E. oleracea* showed improvements in rat testicles affected by cadmium toxicity via upregulated reproductive gene expression and its antioxidant effects.

## Introduction

Infertility stands as a global concern jeopardizing couples worldwide; several factors contribute to about 40%–50% of male infertility, encompassing sluggish sperm motility, a lowered count of sperm, and heightened sperm morphology defects. Lifestyle (smoking and drinking), environmental toxins, genetic variants, and pathological conditions contribute to sperm deoxyribonucleic acid (DNA) fragmentation due to oxidative stress and apoptosis [1]. Environmental toxins are the main causes of infertility; cadmium “Cd” is a hazardous heavy metal in the environment; it is classified as the seventh of the ten general toxic compounds to human health according to the ATSDR ranking [2, 3]. Cd is used in rubber processing, steel plates, batteries, cosmetics, glass, plastic pigments, pesticides, and many industrial products. It is absorbed in large quantities from air pollution, contaminated water, food, soil, and cigarette smoke, causing tissue toxicity such as respiratory, reproductive, skeletal, and cardiovascular toxicity in animals and humans, as well as being considered a carcinogenic substance for humans by an international agency for cancer research [4].

Among the deleterious characteristics of cadmium is that it can accumulate in the liver, heart, kidney, brain, testis, and lungs and alter their functions, so it is considered an accumulative toxicant due to its lower excretion rate and long biological half-life [5,6]. Cadmium can alter the function and structure of the testis, causing testicular degeneration and toxicity that are well described in [6]. Cd can cause damage to the testicular blood barrier and cause the loss of seminiferous epithelia, interstitial cells, and germinal cells, in turn resulting in reduced testosterone levels, impaired spermatogenesis, and impaired sperm functions as a consequence of Cd toxic effects in Sertoli cells and Leydig cells [7].

Several researchers attempted to counteract the testicular toxicity of cadmium, focusing on the natural products and phytotherapy that have antioxidant properties, such as curcumin [8] and *Artocarpus altilis* [9]*. Cyperus esculentus* (tiger nut, nut grass, or chufa sedge) is an herbaceous plant belonging to the Cyperaceae. It is an ancient Egyptian plant; its rhizomes can be grown from tubers and the base of tiger nuts [10]. *C. esculentus* has several beneficial effects due to its potential biological components, such as alkaloids, sterols, flavonoids, polyterpenes, tannins, polyphenols, salicylic acid, terpenoids, and other phytochemical contents [11]. The tubers are considered a good source of vitamins B (B12, B9, B5, B2, B1, and B6) and minerals like iron, sodium, magnesium, calcium, and zinc [12].

Tiger nuts can be used to treat dysentery, indigestion, and flatulence, which extends into interference with colon cancer [12]. Several studies have reported the activity of the aqueous extract of tiger nut on kidney, adrenal gland, liver, and testis functions; this action can be attributed to the large quantities of antioxidants in* C. esculentus,* which exhibit effects in combating hepatic, renal, and testicular problems caused by toxic substances like carbon tetrachloride [13–15]. Furthermore, another study by Udoh et al. [16] illustrated the ability of the aqueous extract of* C. esculentus* to reverse the testicular damage triggered by radiation and enhance spermatogenesis and steroidogenesis [16].

*Euterpe oleracea* fruit, commonly known as an açai, is a potent antioxidant plant due to anthocyanins, phenolic acid, and flavonoids. The pulp and seeds of açai contain various quantities of phytochemicals; açai seeds contain 28.3% polyphenols (oligomeric, polymeric, proanthocyanidins, epicatechin, and catechin), 2% lipids, 5% protein, and 65% fiber [17]. The açai pulp contains 9% anthocyanin, 11% lignoids, 31% flavonoids, and 25.5% polyphenol [17]. Cyanidin-3-O-glucoside and cyanidin-3-O-rutinoside are the major anthocyanins responsible for fruit color [18].

Evidence leans toward supplementing açai, which could suppress reactive oxygen species (ROS, generation in polymorphonuclear cells and reduce cerebral and pulmonary oxidative stress [19,20]. *E. oleracea* has antioxidant activity, which reduces sperm DNA fragmentation and enhances fertility [21].

Studies have investigated the deleterious effects of cadmium chloride (CdCl_2_) on testicular tissues, and the ameliorating effects of *E. oleracea* or CHE when administered individually, as illustrated by Udefa et al. [22] reported the effect of *C*.* esculentus* on sperm functions and the antioxidant activity after exposure to lead acetate; whereas Mouro et al. [23] investigated the antioxidant role of *E. oleracea* on testicular tissues after exposure to cadmium, but their reports do not show their additive effects when given together, as well as those reports do not investigate the possible mechanisms by which both C.* esculentus* and* E. oleracea* can be improved testicular functions at the gene expression level. Based on these limitations, the current study was designed to explore the possible mechanism(s) at the hypothalamic-pituitary-testicular axis by which *E. oleracea* with CHE can improve and enhance testicular functions in CdCl_2_-treated rats.

## Material and Methods

### Ethical approval

The ethical approval for the current experiment was obtained from the ethics committee of Veterinary Medicine at Al-Qasim Green University and approved to guide the care and use of laboratory animals (ESCVM, No. 1192022).

### Preparation of tiger nut hydroethanolic extract

The tubers of *C. esculentus* were obtained from the Babylon market for herbal medicine in Babylon province. The hydroethanolic extract was prepared according to the established methodology [32]. 500 gm of tubers were ground and then dissolved in 5,000 ml of hydro-ethanolic solution (7:3 ethanol: H_2_O) for 48 h at room temperature. The solution was filtered and concentrated by a rotary evaporator at 45ºC and then kept at 4ºC in the refrigerator. The percentage yield of the extract was 163.5 gm.

### *Acute toxicity “LD50” of* CHE

The acute toxicity of the hydroethanolic extract of *C. esculentus*, CHE, was detected according to the Lorke method [24] with two phases. In the initial trial, initial trial (INT) phase, nine male rats were allocated equally into three groups and administered *C. esculentus* extract at doses of 10, 100, and 1,000 mg/kg BW Rats were manifested for 48 h for mortality and any toxicity signs. Depending on the results of the INT stage, the second phase comprises 16th male rats, which are divided into four equal groups and receive orally a graded dose of CHE (1,250, 2,500, 3,750, and 5,000 mg/kg) and monitor the clinical signs and mortality every 4 h for 24 h. This method uses geometric means to determine the lowest lethal dose and the highest nonlethal dose.

### Animals

Adult male Wistar rats aged 12–15 weeks and weighing 160–210 gm were used in the current experiment. They were provided with standard feeding and drinking water *ad libitum* and housed in the well-ventilated standard condition of a 12-h light/dark cycle at 28ºC. This experiment was conducted from September 15, 2022, to May 23, 2023, in the animal house at Veterinary Medicine/Al-Qasim Green University. The rats were allowed to acclimate for two weeks before starting the current experiment.

### Experimental design

Forty mature rats were equally divided into five groups. The control rats were given orally 5 ml of NS to serve as the negative control. Four treated groups were injected intraperitoneally with 1 mg/kg BW of CdCl_2_ in a single dose for two weeks to induce testicular damage, as previously detailed [25]. After two weeks, the second group, “Cd group,” received 5 ml of NS orally as a positive control; the third group, “TC group,” received 800 mg/kg BW of hydroethanolic extract of tiger nut “*C. esculentus*” (CHE, by oral gavage; and the fourth group, “TO,” received 500 mg/kg BW of açai *E. oleracea* (Natrol^®^, USA) dissolved in saline by oral gavage [26]. The fifth group, “TCO group,” received orally 800 and 500 mg/kg BW of *C. esculentus* hydroethanolic extract (CHE) and *E. oleracea*, respectively.

After two months, rats were weighed, and blood samples were collected for hormonal and biochemical parameters such as LH, follicle-stimulating hormone (FSH), testosterone, and CAT determination, then sacrificed. The relative weights of genital organs were measured, and their testicular samples were embedded in formalin 10% for histological study. The hypothalamus and pituitary had been taken to detect the expression levels of *Gnrh, Lhβ, and Fshβ*.

### Body and gonads organ weight measurement

Each rat‘s initial body weight (IW) was calculated at the beginning of the experiment (0 days). Rat‘s final body weight (FW) had been recorded before cervical dislocation, testis, and epididymis weight to body weight were calculated as relative gonad weights.

### Analysis of sperm function

To determine the count of sperm, the epididymal tail was harvested and cut into tiny incisions with 2 ml of saline at 37ºC, and one drop of semen suspension was put in a Neubauer chamber to calculate the count according to a previously outlined protocol [26]. The sperm motility was assessed by putting semen suspension on the slide, covered with a glass slip, and manifested by a light microscope, as described in [28].

The abnormality of sperm was determined using the approach detailed by [29] via placing a drop of epididymal suspension and smearing after that, stained with eosin-nigrosine, then manifested under a microscope. The live and dead sperm were calculated by placing a drop of suspension in the slide, mixed with eosin-nigrosine, and calculated under a microscope; the live sperm heads did not retain stain, whereas those dead were stained [30].

### Determination of hormones, antioxidants, and oxidant parameters

Blood samples were collected at the end of the experiment and centrifuged at 5,000 rpm for 10 min to obtain sera to determine biochemical concentrations [31]. Serum concentrations of LH, FSH, and testosterone were evaluated utilizing the ELISA technique described by manufacturer instruction (AFG Biosceince LLC, Co., US.). In addition, testicular malondialdehyde (MDA), nitric oxide (NO), glutathione peroxidase (GPx), catalase (CAT), and superoxide dismutase (SOD) were measured following established protocols for available kits (AFG Biosceince LLC, Company, US.).

### Total RNA extraction and determination of its purity

Total Ribonucleic acids (RNAs) from rat anterior pituitary and hypothalamus tissues were extracted using a TRIzol^®^ kit (“Bioneer, Korea”). To measure the RNA purity and quantity, a nanodrop spectrophotometer, “Thermos. USA,” was utilized for this purpose.

### cDNA synthesis, preparation of master mix, and analysis of RT-qPCR

To remove the trace quantities of DNA from eluted RNA, the total extracted RNA was treated with the DNase-I enzyme, which was consistent with the instructions explained by Promega Company, USA.

After that, total samples were converted into cDNA; the master mix was prepared depending on the instructions described by “Bioneer Company, Korea.” The primers used in the current study are illustrated in Table 1. SYBER Green dye was used to evaluate the amplification of genes by the real-time PCR machine “Bioneer Company, Korea.” The relative changes (fold changes) of mRNA levels were estimated by ∆∆Ct method as described previously [32].

**Table 1.** Primer sequences were used in the present experiment.

Gene	Primer	Sequence (5’-3’)	References
*Gnrh*	*Forward* *Reverse*	“CCA GCA CTG GTC CTA TGG GT” “AGA GCT CCT CGC AGA TCC CT”	[27]
*Fshβ*	*Forward* *Reverse*	“TTG CAT CCT ACT CTG GTG CT” “AGC TGG GTC CTT ATA CAC CA”	[27]
*Lhβ*	*Forward* *Reverse*	“ATC ACC TTC ACC ACC AGC AT” “GAC CCC CAC AGT CAG AGC TA”	[27]
*Gapdh*	*Forward* *Reverse*	“AAG GTC ATC CCA GAG CTG AA” “ATG TAG GCC ATG AGG TCC AC”	[32]

### Assessment of StARs and steroidogenic enzyme activities

To determine the testicular steroidogenic enzyme activities and StAR levels, approximately 100 mg of testicular tissues were washed and homogenized separately in a solution of phosphate-buffered saline (PBS). Samples were centrifuged at 5,000 g for 5 min in a cold centrifuge at 4ºC. After that, the supernatant was utilized to assess the StAR level, 3β-Hydroxysteroid dehydrogenase (3β-HSD), and 17β-Hydroxysteroid dehydrogenase activities (17β-HSD) according to the manufacturer’s instructions for rats-specific ELISA kits (Elabscience, USA).

### Histological examination

Testis specimens were taken and prepared for histological study in accordance with a previously outlined method [33].

### Statistical analysis

The current data were statistically performed by ANOVA-I and using statistical package for the social sciences (SPSS-16). The current results were presented as mean (M) ± standard deviation (SD). The statistical differences are significant at a level of *p*-value <0.05 [34].

## Results

### Acute toxicity of CHE

The acute toxicity results of the CHE are illustrated in Table 2. It showed that the CHE can be given up to 5,000 mg/kg BW with no vital behavioral signs or mortality in both phases.

**Table 2.** Acute toxicity of *Cyperus esculentus* hydroethanolic extract in rats.

Dose (mg/kg)	Number of rats used	Rats dead/Rats used	Percentage of mortality
Initial phase			
10	3	0/3	**0**
100	3	0/3	**0**
1000	3	0/3	**0**
Second phase			
1250	4	0/4	**0**
2500	4	0/4	**0**
3750	4	0/4	**0**
5000	4	0/4	**0**

### Effects of CHE and E. oleracea on body weight and relative genital weights in male rats exposed to CdCl_2_

Table 3 displays body and relative genital weights in rats exposed to CdCl_2_ in various experimental groups. The IWs appeared to have non-significant (*p* > 0.05) variances among experimental groups that indicate the rats’ weights were matched. The FW, body weight changes (BWG), relative testis weight (RTW), and relative epididymis weight (REW) significantly declined (*p* < 0.05) in rats exposed to CdCl_2_ compared with all experimental groups (Table 3). Interestingly, the FWs and body weight changes were significantly (*p* < 0.05) elevated and enhanced in TC, TO, and in rats receiving CHE with *E. oleracea* in comparison with the Cd group (Table 3).

The relative weights of genitalia were significantly increased (*p* < 0.05) in rats that received an extract of *C. esculentus* with *E. oleracea* in comparison with all experimental groups. Additionally, TC and TO showed a significant (*p* < 0.05) elevation in RTW and REW parameters compared with rats exposed to Cd only (Table 3).

### Effects of CHE and E. oleracea on sperm parameters in adult male rats exposed to CdCl_2_

The sperm motility, count, and viability were significantly inhibited (*p* < 0.05) in rats exposed to cadmium compared to all experimental groups (Table 4). Conversely, these parameters recorded a significant (*p* < 0.05) raise and enhancement in the TCO group compared with all experimental groups. The percentages of abnormal morphology of sperm and dead sperm were significantly (*p* < 0.05) the highest in the Cd group compared with other experimental groups. In contrast, these latter parameters declined significantly (*p* < 0.05) in the TCO group compared with all experimental rats (Table 4).

### Effects of CHE and E. oleracea on testicular oxidative stress and antioxidant status in adult male rats exposed to CdCl_2_

**Figure 1.**
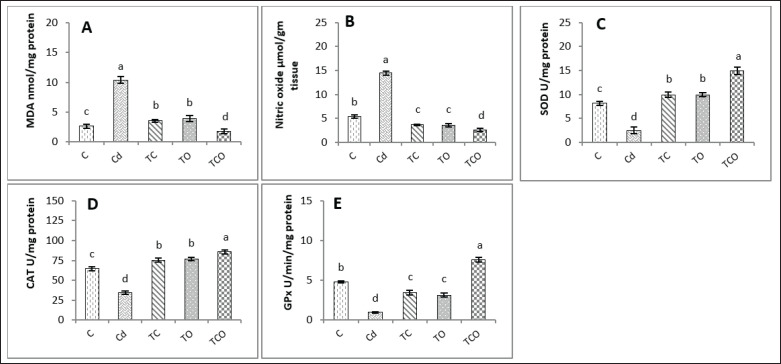
Testicular oxidative stress and antioxidant markers in rats. Dissimilar letters refer to significant differences (*p* < 0.05) among groups. C: control group, Cd: 1 mg/kg BW of cadmium chloride, TC: Cd+ *C. esculentus* extract (800 mg/kg BW), TO: Cd+ *E. oleracea* (500 mg/kg BW), TCO: Cd+ *C. esculentus* extract with *E. oleracea* 800 and 500 mg/kg BW, respectively. (A) MDA: malondialdehyde. (B) NO: Nitric oxide. (C) SOD: superoxide dismutase. (E) CAT: catalase. (E) GPx: glutathione peroxidase.

The current results showed significant (*p* < 0.05) elevations in testicular MDA (tMDA) and tNitric Oxide levels in the Cd rats compared with the TC, TO, TCO, and control groups. When compared with all experimental groups, the TCO rats recorded a decline (*p* < 0.05) in testicular MDA and nitric oxide levels (Fig. 1A and B, respectively). Interestingly, the statistical analysis of current data revealed there was a significant (*p* < 0.05) elevation in testicular SOD (tSOD) and testicular CAT (tCAT) activities in TCO compared with other treatment groups (Fig. 1C and D). tSOD and tCAT recorded a significant (*p* < 0.05) raise in TC and TO in comparison with the Cd and control groups; the Cd group recorded a significant (*p* < 0.05) drop in these parameters compared with all experimental rats.

**Table 3.** Effects of *Cyperus esculentus* hydroethanolic extract and *Euterpe oleracea* on body weight and relative genital weights in adult male rats exposed to CdCl_2_.

Parameters	C	Cd	TC	TO	TCO
IW (g)	162.86 ± 4.02^a^	164.96 ± 3.899^a^	164.54 ± 4.75^a^	162.7 ± 3.99^a^	163.8 ± 5.357^a^
FW (g)	226.08 ± 3.26^b^	117.2 ± 5.019^d^	217.6 ± 1.673^c^	216.6 ± 3.782^c^	247.54 ± 2.933^a^
BWG (g)	63.22 ± 4.486^b^	-47.76 ± 4.415^d^	53.06 ± 5.591^c^	53.9 ± 5.249^c^	83.74 ± 3.95^a^
RTW (%)	1.751 ± 0.046^b^	0.682 ± 0.147^d^	1.566 ± 0.02^c^	1.507 ± 0.038^c^	2.51 ±0.129^a^
REW (%)	0.5142 ± 0.056^b^	0.076 ± 0.011^d^	0.341 ± 0.019^c^	0.335 ± 0.033^c^	0.967 ± 0.18^a^

Values represented mean ± standard deviation. Dissimilar letters refer to significant differences (p < 0.05) among groups. C: control group, Cd: 1mg/kg b.w. of cadmium chloride, TC: Cd+* C. esculentus* hydroethanolic extract (800mg/kg b.w.), TO: Cd+* E. oleracea* (500mg/kg b.w.), TCO: Cd+* C. esculentus* hydroethanolic extract with* E. oleracea* 800 and 500 mg/kg b.w., respectively, IW: initial body weights, FW: final body weights, BWG: body weight changes, RTW: relative testis weight, REW: relative epididymis weight.

**Table 4.** Effects of *Cyperus esculentus* hydroethanolic extract and *Euterpe oleracea* on sperm parameters in adult male rats exposed to CdCl_2_.

Parameters	C	Cd	TC	TO		TCO
Sperm count (million/ml.)	47.994 ±0.707^b^	22.98 ± 1.184^d^	41.80 ± 1.303^c^	41.54 ± 0.953^c^	64.94 ± 0.639^a^	
Sperm motility (%)	73.40 ±2.408^b^	34.60 ± 3.435^c^	71.60 ± 1.14^b^	71.0 ±1.224^b^	89.00 ±1.870^a^	
Live sperm %	71.50 ±3.162^b^	32.70 ± 4.35^d^	68.82 ±3.15^bc^	65.80 ± 4.816^c^	88.10 ± 1.51^a^	
Dead sperm %	28.5 ±3.162^c^	67.3 ± 4.35^a^	31.18 ± 3.156^bc^	34.2 ± 4.816^b^	11.9 ± 1.517^d^	
Abnormal morphology (%)	9.36±0.321^c^	38.62 ± 1.837^a^	11.48± 0.37^b^	11.98 ± 0.609^b^	7.24 ±0.929^d^	

Values represented mean ± standard deviation. Dissimilar letters refer to significant differences (p < 0.05) among groups. C: control group, Cd: 1mg/kg b.w. of cadmium chloride, TC: Cd+* C. esculentus* hydroethanolic extract (800mg/kg b.w.), TO: Cd+* E. oleracea* (500mg/kg b.w.), TCO: Cd+* C.esculentus* hydroethanolic extract with* E. oleracea* 800 and 500 mg/kg b.w., respectively.

Testicular GPx (tGPx) activity exhibited a significant (*p* < 0.05) increment in TCO rats compared with all experimental groups. The results of tGPx activity reported a significant (*p* < 0.05) decline in Cd rats compared with all treatment groups (Fig. 1E).

### Effects of CHE and E. oleracea on reproductive hormones in adult male rats exposed to CdCl_2_

**Figure 2.**
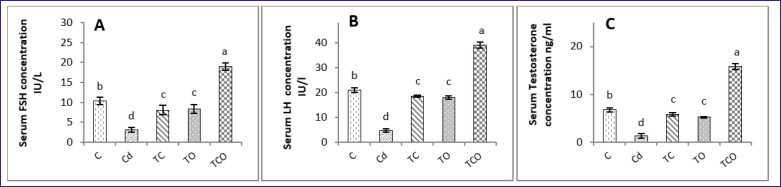
Serum (A) FSH, (B) LH, and (C) testosterone concentrations in experimental groups. Dissimilar letters refer to significant differences (*p* < 0.05) among groups. C: control group, Cd: 1 mg/kg BW of cadmium chloride, TC: Cd+ *C. esculentus* extract (800 mg/kg BW), TO: Cd+ *E. oleracea* (500 mg/kg BW), TCO: Cd+ *C. esculentus* extract with *E. oleracea* 800 and 500 mg/kg BW, respectively.

The levels of serum FSH, LH, and testosterone were significantly (*p* < 0.05) elevated in the TCO group in comparison with the Cd, TC, TO, and control groups, whereas their concentrations were significantly (*p* < 0.05) lowered in the Cd group compared with other treatment groups. FSH, LH, and testosterone levels were enhanced significantly (*p* < 0.05) in TC and TO rats compared with the Cd rat (Fig. 2A–C).

### Effects of C. esculentus hydroethanolic extract and E. oleracea on fold change of hypothalamic Gnrh and pituitary Fshβ and Lhβ gene expression in adult male rats exposed to CdCl_2_

**Figure 3.**
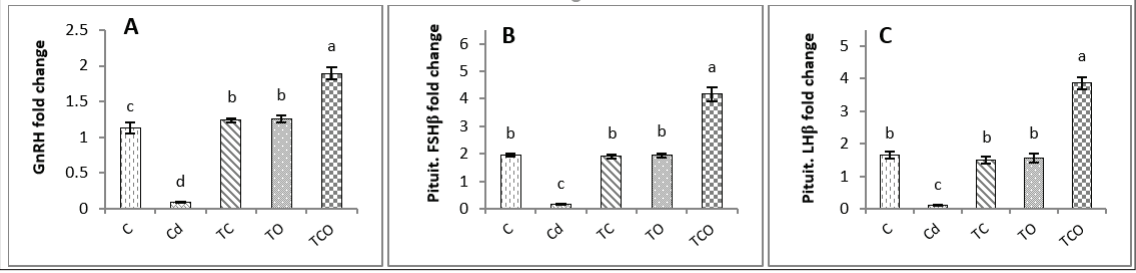
Hypothalamic and pituitary change fold in experimental rats. (A) Hypothalamic GnRH change fold. (B) Pituitary FSHβ change fold. (C) LHβ change fold. Dissimilar letters refer to significant differences (*p* < 0.05) among groups. C: control group, Cd: 1 mg/kg BW of cadmium chloride, TC: Cd+ *C. esculentus* extract (800 mg/kg BW), TO: Cd+ *E. oleracea* (500 mg/kg BW), TCO: Cd+ *C. esculentus* extract with *E. oleracea* 800 and 500 mg/kg BW, respectively.

The fold change of hypothalamic *Gnrh*, *Fshβ*, and *Lhβ* gene expressions was significantly (*p* < 0.05) higher in TCO rats than in other experimental rats. The fold of these gene expressions was elevated (*p* < 0.05) in TC and TO compared to the Cd group. Whereas *Fshβ*, *Gnrh*, and *Lhβ* were significantly suppressed (*p* < 0.05) in rats exposed to CdCl_2_ compared to all experimental groups (Fig. 3A–C).

### Effects of C. esculentus hydroethanolic extract and E. oleracea on StAR steroidogenic enzyme activities in adult male rats exposed to CdCl_2_

**Figure 4.**
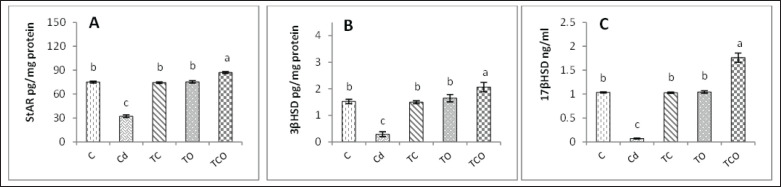
Testicular level of StAr protein and steroidogenic enzymes activities in experimental rats. (A) StAR: Sterioidogenic acute regulatory protein. (B) 3β-HSD: 3β-Hydroxysteroid dehydrogenase. (C) 17β-HSD:17β-Hydroxysteroid dehydrogenase activities. Dissimilar letters refer to significant differences (*p* < 0.05) among groups. C: Control group, Cd: 1 mg/kg BW of cadmium chloride, TC: Cd+ *C. esculentus* extract (800 mg/kg BW), TO: Cd+ *Euterpe aleracea* (500 mg/kg BW), TCO: Cd+ *C. esculentus* extract with *E. oleracea* 800 and 500 mg/kg BW, respectively.

In the Cd-treated group, the testicular level of StAR protein and the activities of 3β-hydroxysteroid dehydrogenase (3β-HSD), and 17β-Hydroxysteroid dehydrogenase activities (17β-HSD) were significantly (*p* < 0.05) declined relative to TCO, TO, TC, and control groups (Fig. 4A–C). Intriguingly, the StAR protein level and steroidogenic enzyme activities were significantly (*p* < 0.05) increased in the TCO group compared to the TO, TC, Cd, and control groups (Fig. 4A–C), respectively.

### Histopathological changes

**Figure 5.**
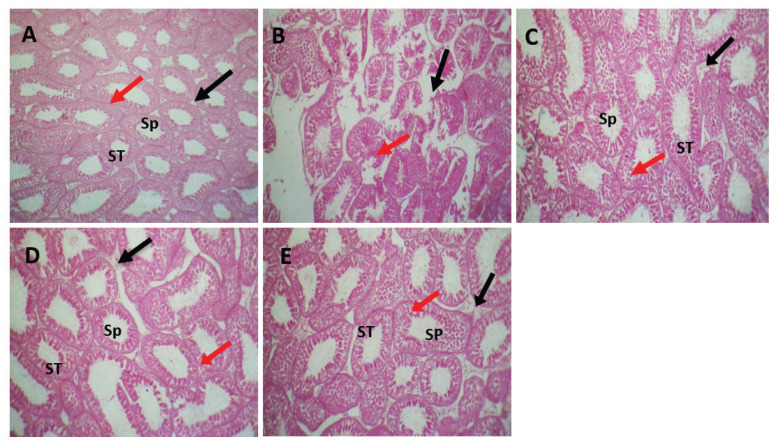
Testicular photomicrographs of all experimental rats. (A) control group: showed a normal feature of seminiferous tubules with normal Leydig and Sertoli cells with series spermatogenesis. (B) Cd group: showed sever damaged in seminiferous tubules and degenerative changes with loosing spermatogonia, and Sertoli cells after treated with cadmium chloride. (C) TC group (Cd+* C. esculentus)*: showed seminiferous tubules return to the normal texture (regenerative process) and occupied with few spermatozoa after treated with 800 mg/kg BW of hydroethanolic extract of *C. esculentus*, and Leydig cells return to normal feature with normal Sertoli cells and germinal epithelia. (D) TO group (Cd+* E. oleracea)*: showed that the seminiferous epithelia back to normal feature and filled with spermatozoa, with normal Leydig and Sertoli cells. (E) TCO group (Cd+* C. esculentus* extract with* E. oleracea*): showed a normal texture of seminiferous tubules occupied with spermatozoa with normal Leydig and Sertoli cells and complete active spermatogenesis, H&E 40X. Seminiferous tubules (ST), spermatozoa (Sp), Leydig cells (black raw) and Sertoli cells (red raw).

The testicular sections of the control group appeared to have a typical architecture without histological alteration (Fig. 5A), with the normal appearance of seminiferous epithelia, Sertoli cells, and interstitial cells with series spermatogenesis recorded. On the contrary, the testicular sections of rats exposed to cadmium showed congestion in interstitial capillaries with damage to the seminiferous tubules and Sertoli cells, with degenerative changes accompanied by the complete absence of spermatozoa and reduced spermatogenesis in degenerated tubules (Fig. 5B). The testicular sections of rats receiving cadmium and *C. esculentus* or *E. oleracea*, TC, and TO groups revealed that the seminiferous tubules return to normal appearance with moderate spermatogenesis (Fig. 5C and D). The testicular sections obtained from the TCO group appear to have a typical architecture similar to that shown by control rats (Fig. 5E), with a typical structure of seminiferous tubules and interstitial cells. Primary and secondary spermatocytes with series spermatogenesis were evident.

## Discussion

The current experiment has been carried out to compare and evaluate the additive effects of the hydroalcoholic extracts of *C. esculentus* and *E. oleracea* on CdCl_2_-induced testicular degeneration in adult male rats. The present study showed that exposure to CdCl_2_ caused a lowering in FW, body weight changes, as well as the relative weight of the epididymis and testis, due to the ability of CdCl_2_ to suppress the digestion process and absorption via inhibition of the digestive enzymes [35], which in turn reduced feed efficacy and nutrient absorption. Additionally, CdCl_2_ can induce oxidative stress and cellular DNA damage that negatively reflects on body weight and testicular and epididymal relative weight [35].

However, the current results found a significant enhancement in FWs, body weight changes, testis, and epididymal relative weights in TC, TO, and TCO rats that can be attributed to the active biological components of each *E. oleracea* and/or* C. esculentus* can ameliorate the adverse effects of CdCl_2_ via protecting cells membrane and enhancing functional and structural proteins biosynthesis causing to improve body organs functions [21,36], previous studies reported elevation in body weight changes resulting from enhanced the appetite, food conversion rate, and some digestive enzymes like amylase and lipase in rats give *C. esculentus* extract/or oil and *E. oleracea* [21,36]. Additionally, these effects in TCO rats might be attributed to an elevation of androgen synthesis that significantly affects testicular and epididymal relative weights in rats, which agrees with previously reported results [19,37].

Sperm is sensitive to ROS production due to its membrane components of polyunsaturated fatty acids, leading to sperm dysfunctions, decreased sperm motility, and infertility; this is in line with another study that has concluded that CdCl_2_ caused defects in sperm production, morphology, viability, and decline in steroidogenic and antioxidant enzymes [38] via induced oxidative stress and lipid peroxidation leading to damage in protein and DNA of sperm, degeneration and damaged of testicular cells [39], the current results were consistent with prior research [40] who found a decline in sperm functions after exposure to CdCl_2_ for 15–30 days which occurred due to generate large quantities of ROS that reduced antioxidants synthesis thereby resulting in lipid peroxidation in testicular tissues causing harmful effects on membrane integrity of spermatogonia and spermatozoa and spermatogenesis disruption [40].

At the same time, accordance to current results, *C. esculentus* hydroethanolic extract with *E. oleracea* “CHE-EO” can demonstrate an additive effect at the specified dosages; however, their effectiveness is evident when administered individually in comparison to the control, due to their abilities to improve the sperm functions and reduce the adverse effects of exposure to CdCl_2_ as reduced the dead and abnormal sperm with increasing motility, count, live sperm and decrease abnormal sperm in CdCl_2_-CHE-EO- treated rats that contributed to the ability of anthocyanin, major natural antioxidant of *E. oleracea*, to lower CdCl_2_ accumulation in blood and testis, suppress the oxidative stress and reduce the sperm deformities, that consistent with finding of [37], as well as, administrated *C. esculentus* extract* with E. oleracea* can enhance and improve the testicular antioxidant defense system which aid in protection of cell membranes from oxidative damage due to their contents from quercetin, zinc, vitamins E and C that protect DNA of sperm from ROS and oxidative stress [41], besides, *E. oleracea* with *C. esculentus* extract could induce the signal alteration between spermatozoa and calcium that improve sperm movements [22,23] that positively affect sperm motility in TCO treated rats.

The statistical analysis of current results recorded a significant elevation in tMDA and nitric oxide “tNO” with a reduction in testicular SOD, GPx, and CAT in rats administered CdCl_2_, which is regarded as a toxic damage marker in testis. These results align with the previous study [7], which attributed the ability of CdCl_2_ to damage cell membranes, causing pathological alteration and oxidative stress resulting from excessive production of ROS and nitrogen radicles, which in turn impaired the DNA, proteins, and lipids of testicular cells [40].

Cadmium can cause testicular toxicity via GSH exhaustion, and chelating proteins contain amino groups or hydroxyl and sulfhydryl groups; moreover, CdCl_2_ can alter protein biosynthesis by competing and displacing the essential minerals and metals, resulting in lipid peroxidation leading to an increasing MDA level [8]. The elevation of testicular NO in rats receiving CdCl_2_ results from an upregulated eNOS level that affects the biosynthesis of antioxidants, suppresses synthesis, and releases testosterone from Leydig cells [39]. In current results, the activities of tSOD, tGPx, and CAT declined in rats exposed to CdCl_2_ due to the ability of Cd to interact with iron, which led to iron deficiency because iron is considered a structural building block of the CAT active site. Besides, CdCl_2_ can interact with divalent ions such as zinc, copper, and manganese, which led to a decline in testicular SOD [40].

Intriguingly, the current results revealed a significant elevation in the activities of the antioxidant enzyme in rats that received both *C. esculentus* hydroethanolic extract and *E. oleracea,* which may be attributed to their ability to induce the synthesis of the antioxidants with diminished NO and MDA levels, as well as the additive effects of phytochemicals in *C. esculentus* hydroethanolic extract and *E. oleracea* like flavonoids, anthocyanins, quercetin, phenolic acid, vitamin C, and vitamin E that potentially enhance the antioxidant system in rats [21,42].

Furthermore, the phenolic compounds in these extracts can act as a donor of one atom of hydrogen or an electron to neutralize the reactive species, reducing oxidative stress, MDA, and NO levels [43]. A recent study [44] reported the role of cyanidin-3-O-glucoside and cyanidin-3-O-rutinoside of açai hydroethanolic extract to upregulate CAT, GPx, and SOD, in turn reducing ROS and reactive nitrogen species (RNS).

Cadmium exposure in rats caused a significant decline in LH, FSH, and testosterone concentrations, suppressed the levels of the pituitary *Fshβ, Lhβ* gene, and hypothalamic *Gnrh* gene, and caused histopathological alteration of testicular tissues. A decrease in these hormone concentrations is an indication of spermatogenesis disruption. CdCl_2_ is considered a toxic substance for the brain; it accumulates in pituitary and hypothalamic tissues, causing deformity and damage in hypothalamic and pituitary cells, leading to disruption of gene expression of pituitary *Gnrhr, Fshβ,* and *Lhβ*, and hypothalamic *Gnrh* [41], thus affecting FSH and LH secretion as observed in Cd exposure rats (Fig. 2A and B). The drop-in serum FSH concentration in rats exposed to CdCl_2_ could have been causing a nutrient deficiency that is essential for sperm development and, in turn, suppresses spermatogenesis and live sperm, as observed in Cd rats [9]. Testosterone released from Leydig cells is necessary for the proliferation and growth of testicular germ cells; a decline in testosterone secretion is evidence of male reproductive toxicity [40].

Testicular toxicity, as found in histopathological changes and a reduction in testosterone level after exposure to CdCl_2_, could be a reason for the decrease in spermatogenesis and sperm count [38]. In addition, Iqbal and colleagues [35] reported that cadmium caused disorganization of mitochondrial Leydig cells, a decrease in the number of Leydig cells, and damage to DNA Leydig cells, causing testicular degeneration and a decrease in testosterone secretion, as seen in Figure 5B. As well, exposure to CdCl_2_ can suppress the expression of *Cyp11a1, Scarb1, StAR, Hsd17b, Hsd1b1, Hsd17a1, Hsd3b,* and *Lhcgr,* which lowers testosterone levels [38].

Interestingly, upon the co-administration of CdCl_2_ and *C. esculentus* hydroethanolic extract with *E. oleracea*, the levels of reproductive hormones increased significantly compared with Cd rats due to the ability of *C. esculentus* hydroethanolic extract to induce gene expression of pituitary *Lhβ, Fshβ,* G*nrhr, and* hypothalamic* Gnrh* resulting in elevating the levels of LH and FSH which effected on Leydig cells and Sertoli cells to stimulate testosterone synthesis and spermatogenesis, respectively [37], as well as, *C. esculentus* hydroethanolic extract can upregulate 17β-HSD, 3β-HSD and steroidogenic acute regulatory protein (StAR) in testicular tissues, in turn, elevated testosterone levels [16,45], this effects might be attributed to the extract phytochemical components from trace elements such as zinc and vitamin C and E and antioxidant such as quercetin [46], that ameliorated the Leydig and Sertoli cells function as proved by current study (Fig. 4).

Besides, *E. oleracea* can recover seminiferous epithelium and normalize the Leydig cells, which is attributed to the ability of *E. oleracea* constituents to upregulate the StAR proteins *3β-HSD, 17β-HSD* and *CYP11A1* in Leydig cells that facilitate steroidogenesis and, in turn, increase testosterone levels (Fig. 4) [23,46]. Interestingly, the biological constituents of both *C. esculentus* hydroethanolic extract and *E. oleracea* act as potent factors for upregulating StAR protein and steroidogenic enzymes that positively affect spermatogenesis and androgen biosynthesis, which ameliorates histological changes in the TOC group.

## Conclusion

The results reported in this study revealed a potential role of *E. oleracea* with a hydroethanolic extract of *C. esculentus* to treat the damage to testicular tissues induced by CdCl_2_ via ameliorating the antioxidant activity that improved the hypothalamic-hypophyseal-testis axis, resulting in upregulated gene expression of reproductive hormones and improved testicular steroidogenesis and spermatogenesis. Therefore, several studies are required to investigate the roles of* E. oleracea* and *C. esculentus* on gene expression of thyroid hormones in hypothyroidism and hyperthyroidism in male and female rats. This study didn’t report the anti-inflammatory effects of both *E. oleracea* and the hydroethanolic extract of *C. esculentus*, as well as their effects on gene expression of inhibin and activin hormones.

## List of abbreviations

BWG, body weight changes; Cat, catalase; CdCl_2_, cadmium chloride; Cd group, 1 mg/kg BW of cadmium chloride; CHE,* Cyperus esculentus* hydroethanolic extract; DNA, Deoxyribonucleic acid; FSH, Follicle-stimulating hormone; FW, final body weights; GPx, glutathione peroxidase; 17β-HSD, 17β-hydroxysteroid dehydrogenase; 3β-HSD, 3β-hydroxysteroid dehydrogenase; INT, initial trial; IW, initial body weights; MDA, malondialdehyde; NO, Nitric oxide; REW, relative epididymis weight; RNA, Ribonucleic acid; RTW, relative testis weight; tSOD, testicular superoxide dismutase; SPSS, Statistical Package for the Social Sciences; StAR, steroidogenic acute regulatory protein; TC, Cd+ *Cyperus esculentus* hydroethanolic extract (800 mg/kg BW); TCO, Cd+ *Cyperus esculentus* extract with *E. oleracea* 800 and 500 mg/kg BW, respectively; TO, Cd+ *Euterpe oleracea* (500 mg/kg BW).
